# A Treatise on the Structure, Diseases, and Injuries of the Blood-Vessels

**Published:** 1847-10

**Authors:** 


### Art. XI.

*A Treatise on the Structure, Diseases, and Injuries of the Blood-Vessels.* By Edwards Crisp.—*London*, 1847. 8vo, pp. 354. With Plates.

The subject selected by the Council of the Royal College of Surgeons for the Jacksonian Prize for 1844, was "the Structure and Diseases of the larger Blood-vessels." Mr, Crisp was the successful competitor, and the present treatise contains the substance of his Prize Essay. More than two years, however, having elapsed since this was written, Mr. Crisp states that he has been enabled by personal observation and the recorded experience of others, to obtain much additional information. He has likewise taken a more comprehensive view of the subject, and treated of injuries as well as diseases of the blood-vessels; in this way the work has been so much enlarged, that he says it is due to the adjudicators to state that they are not in any way responsible for any errors that may have occurred by the introduction of new matter. In treating of the structural and pathological conditions of the blood-vessels, Mr. Crisp states that he has given the results of his own observations, and has not drawn any inferences without careful investigation; and that, being of opinion that the statistical is the only sure method of arriving at accurate deductions in medical inquiries, he has constructed a table of all the cases of spontaneous aneurism that have been reported in the British Journals from the year 1785 up to the present time. The analysis of this table, comprising 551 cases, furnishes the basis of the greater part of our author's remarks on aneurism. The work likewise contains the histories of cases bearing on the subject of which it treats, that have either occurred in the author's practice, or that of his friends, or that have been seen by him at the Borough Hospitals. We shall now proceed to lay before our readers an analysis of the more novel or interesting matters contained in the work itself.

*Structure of Arteries and Veins.* Mr. Crisp, in his views of the structure of arteries, differs from Henle in only recognising four instead of six coats; viz. 1, the internal or serous; 2, the sub-serous, or tunica cellulosa of Haller, or the sclerous of Malgaigne; 3, the fibrous; and lastly, the external cellular. Mr. Crisp has not been able to determine the existence of the epithelial lining, and the broad longitudinal fibres as described by

Henle, from whom he likewise differs in not admitting the muscularity of arteries, or the existence in them of fibres analogous to the unstriped muscular fibre of organic life. He confesses, however, very candidly that his experience in the use of the microscope is not such as to entitle his opinion to much weight. And certainly, putting all microscopical evidence aside, physiological facts are strongly in favour of the correctness of Henle's views, which, in a remarkable manner, bear out John Hunter's opinions with regard to the irritability of the smaller arteries. Mr. Crisp is in error where he says that most anatomists now believe, in opposition to the views of John Hunter, Sömmering, John Thomson, and others, in the non-muscularity of arteries. This may be the case with the larger vessels, to which Mr. Crisp appears principally to have directed his attention, but is certainly not the prevailing opinion with regard to the smaller arterial ramifications. Henle has shown that the arterial tunic composed of flat, clear, granular fibres, resembling the muscular fibres of organic life, is peculiarly developed in the smaller arteries, which possess a distributive power over their contents, and which Hunter proved to be endowed with irritability, whereas the genuine yellow elastic tissue is principally met with in the larger vessels, which are distinguished by their elasticity, which is necessary to sustain the force of the heart's action where it is strongest, and which gradually decreases with the size of the artery.

Mr. Crisp relates some experiments that he has performed upon the arteries of man and the lower animals, with the view of ascertaining the amount of physical elasticity which they possess, the following are the conclusions to which he arrives:

"1. That the fibrous coat of the arteries and veins is highly elastic; that it possesses no properties analogous to muscular fibre; that this tissue differs from all other structures of the body, except the ligamentum nuchæ and ligamentum subflavum, as regards its amount of elasticity.

"2. That the elastic property is the same in every part of the fibrous coat of the larger arteries.

"3. That the arteries of women are rather more elastic than those of men; and that the elastic property generally diminishes after the age of forty-five or fifty.

"4. That the elasticity of an artery decreases with its size.

"5. That the veins are in a slight degree less elastic than the arteries.

"Lastly. That the cellular coat of an artery possesses but little, if any, elasticity." (p. 7.)

The next chapter is devoted to various *Morbid conditions of the Arteries.* The principal question discussed, is "How are the different forms of redness to be distinguished?" On the solution of this question, our author throws no new light whatever. He adopts the usual division of these discolorations into the inflammatory, the congestive, and redness from imbibition, but does not appear to draw any distinction between the two last-named kinds of discoloration, which are no doubt, as Gendrin has proved, merely the result of post-mortem changes in patients whose energies have, during life, been depressed, and in whom the blood is in a morbid condition.

*Arteritis.* Our knowledge of this disease is as yet imperfect. For this there are two reasons: first, the great difficulty of distinguishing inflammatory redness of the serous and sub-serous coats from simple imbibition; and, secondly, the obscurity that, in the majority of cases, attends its symptoms during life. After giving, in a cursory and not very complete manner, the general cause and varieties of this disease, the author proceeds to consider it as it affects the aorta and as it attacks the arteries of the extremities.

If some degree of obscurity shrouds the subject of inflammation of the arteries in general, it is increased when we examine this disease as affecting *the aorta;*—the products of inflammation—lymph, pus, and coagula—being but seldom found in that vessel, and thickening of its coats not being readily appreciable except when extending to the valves. This obscurity, presented by the symptoms of aortitis, has been well pointed out by Dr. Hope, who has shown that it is almost impossible to recognise it during life as an abstract disease, on account of the invariable complication of endocarditis, by which its symptoms are, to use his expression, absorbed. Indeed many of the so-called cases of aortitis on record will prove, on a closer examination, merely to be instances of endocarditis—of this description, for example, are the cases mentioned by Bertin. Mr. Crisp mentions having seen three cases of what he believes to be aortitis, but as only one of these patients died, and in him no post-mortem examination was made, but little value can be placed in them. A table is given of seventeen cases in which the aorta was obliterated during life; on looking over the details of these, it is curious to observe how little inconvenience most of the individuals suffered from this abnormal condition of the aorta. One man lived to the age of 93, and four others died above the age of fifty. Two patients died from rupture of the ascending aorta, another from rupture of the left ventricle, and a fourth from rupture of the right auricle. In the greater number of the cases, the aorta was obliterated or contracted near the point at which the ductus arteriosus opened into it. This liability to obliteration or contraction of the vessel at this part, was first pointed out by Dr. Craigie, and is well illustrated by the cases Mr. Crisp has collected.

*Inflammation of the arteries of the extremities.* In these cases the diagnosis, although obscure, is not so difficult as when the vessel is situated so deeply as the aorta is. There are pains of a burning, pricking, or shooting character, with great tenderness on pressure along the course of the affected vessel. These symptoms are often followed by a cessation of pulsation in the inflamed artery, and a tendency to gangrene, especially when occurring in the lower limbs. In some cases the vessel is hard and cord-like, and there is always more or less febrile excitement. Mr. Crisp has never met with inordinate pulsation as described by some authors. In old people the inflammation is most generally subacute, the pain less severe, the pyrexia not so great, but the tendency to gangrene more manifest. Mr. Crisp is a firm advocate of the doctrine that the gangrene of the legs of old people is the result of arteritis, and gives numerous cases in support of this opinion.

"Some high authorities believe that arteritis is not present in the dry gangrene of old people. I have paid much attention to this complaint for many years, and have had an opportunity of inspecting the bodies of several who have died of it. I have invariably found the lining membrane of the artery of a red appearance, the vessel often obstructed by fibrinous coagulum, some distance above the gangrenous part, as well as in the arteries near the seat of the disease. The inflammatory redness is not present in the preparations alluded to, there is, however, I think, abundant evidence to show that it existed........

"How often the aged are affected with cutaneous inflammation of the legs, and, reasoning from analogy, why, let me ask, may not the internal coats of the arteries be subject to the same engorgements? We have, I think, abundant evidence to show that ossification alone is not sufficient to produce the disease, as this deposit exists to a great extent in the majority of individuals who have attained an advanced age. The most common of the exciting causes of acute arteritis in the aged is, I believe, the detachment of a portion of the bony plate of the artery. The great toe, a part the most exposed to mechanical injury, is often the first affected. This accident I conceive, may occur at any part of the vessel, and its effect will probably be the production of inflammation, plugging of the collateral branches, and the consequent death of the parts below the seat of obstruction." (pp. 33-4.)

It may, however, be questioned whether this deposition of fibrine, and evidence of inflammatory action within the vessels, is a proof that the primary disease begins in them. May it not, in many cases, be the consequence of an obstruction to the passage of the blood through the capillaries. The death in the extreme parts being necessarily followed by deposition of fibrine in the larger vessels leading to those parts. In all cases of mortification, from whatever cause, those vessels that lead to the sphacelated part are blocked up by fibrinous exudation, as a safeguard against the occurrence of hemorrhage in the separation of the sloughs, and are usually found to present more or less of that inflammatory action, which has given rise to the deposition of lymph, and the obliteration of the vessel.

Mr. Crisp's account of *the treatment* of arteritis is somewhat unsatisfactory; but we know not that it could be otherwise. When the disease affects young subjects, he recommends local bloodletting and mercurials. In our opinion, the latter are very badly borne indeed in these cases, unless combined with large doses of opium. In old people our author recommends opium, and a mild unstimulating diet, with attention to the position of the limb. Hot fomentations, linseed meal, yeast, or beer-ground poultices. He makes no mention of a local application that is now very generally employed, and which we have always found much more cleanly and efficient than any moist application, namely, enveloping the limb in carded wool, or cotton wadding, retained by means of a silk handkerchief, and not opened more frequently than is absolutely necessary, in order to dress the sloughy or ulcerated parts.

The following chapters on "*Arterial deposits,*" and on the "*Diseases of the Aortic Valves,*" contain very little that is new or of any interest; we shall accordingly pass them by.

*Abdominal pulsation.* These cases, the author states, may very conveniently be divided into three classes. The first are those depending upon constitutional causes, such as chlorosis, hysteria, anemia from loss of blood, &c., in short, any condition of the system inducing an impoverished condition of the circulating fluid, or derangement of the nervous functions. In the second variety, mechanical obstruction appears to be the chief cause of the pulsation. The most frequent of the mechanical causes, are tumours of various kinds pressing upon the vessel, such as enlarged pancreas or spleen, scirrhous stomach, diseased mesenteric glands, collections of air and scybalous matter in the bowels. Allan Burns likewise mentions solidification of the lower lobes of the lungs, dilated heart, and enlarged vena cava. In the last, and, according to Mr. Crisp, the most frequent form, the aorta appears to be sympathetically affected. Dr.

Baillie supposed that this depended upon a disordered state of the digestive organs, and that it occurred more frequently in the male sex. Mr. Crisp, however, finds that this opinion is incorrect, as the majority of recorded cases have occurred in females. Mr. Crisp agrees with Drs. Fausset and Stokes, that stomach or intestinal affection is generally the exciting cause of this malady; yet he believes that it can only take place when the parietes of the vessel are in a weakened condition; this probably depending upon structural defect, or the derangement of nervous influence.

"It is difficult to imagine that local pulsation can arise from nervous excitability alone, or that the upper part of the aorta can be morbidly excited without the lower portion being in a similar state; if, however, the coats of the artery, from want of innervation or structural peculiarity lose their tone and possess greater tenuity and expansibility in certain parts, the phenomenon is more readily explained." (p. 97.)

The diagnosis of these cases from aneurism, is sometimes attended with difficulty, especially so in those cases in which the pulsation is limited to a small portion of the arterial tube; on this subject, however, our author adds nothing to the signs that had been pointed out by Dr. Hope some years ago, and omits to mention the very valuable diagnostic symptoms to which Dr. Graves was the first to direct the attention of the profession, namely, that the murmur will sometimes occur in the horizontal position in these cases, when from hydrostatic pressure it ceases in the erect.

Aneurism. The section devoted to this subject is the most important and complete of the work. As has already been stated, the materials of which this portion of the treatise is composed, are derived from the analysis of a table of 551 cases of aneurism that have been reported in the British Journals during the last sixty years. After making a few general remarks on the nomenclature, divisions, and comparative frequency of aneurism in different situations, our author proceeds to a consideration of the causes of this disease.

"It will naturally be asked why aneurism is more frequent in one artery than in another? But if we take into account the form, size, and anatomical bearings of the different arteries, the matter I think admits of a ready explanation. Thus, the ascending and right side of the aorta is the most common seat, and here the impulse of the blood is the strongest; especially during sudden and violent contractions of the ventricle. Next in order, as to frequency, comes the arch of the aorta, a part also subject to the blood's impulse, and probably somewhat weakened by the giving off of the large branches; the disease does not so often occur in the descending thoracic, and when it does, it is generally immediately below the arch where the parietes of the vessel are more acted upon by the current of the blood than in the straight portion. In the abdominal aorta, the most common situation is near the cæliac axis, where several large branches are given off, and below, which part the aorta diminishes in size. The common iliac, which is straight and gives off no branches, is rarely affected; and the same remark applies to the external and internal iliac, the branches from the former are very small, and the latter vessel is short and terminates in numerous divisions. The upper portion of the femoral artery is often affected with aneurism. The branches arising from it, the frequent motion of the thigh, and the greater liability of the vessel in this situation to external violence, are probably the chief causes. The middle third of the femoral is seldom subject to this disease, whilst the popliteal, which forms a curve over the joint, gives off several articular branches, is much exposed to violence, and probably to elongation in sudden extension of the leg, is very frequently aneurismal. The disease appears to occur in the branches of the arch in nearly similar proportions; but those on the right side are rather more subject to it. It is difficult to assign a reason for the exemption of the brachial artery from this lesion. The most likely is the looseness of its connexions, and the curvature of the axillary artery, which allows of a certain amount of elongation, and thereby prevents the stretching which probably occurs under certain circumstances in the popliteal. In reviewing the foregoing localities of aneurism, this inquiry naturally presents itself, viz. are the arteries in these situations more subject to disease? After a very careful investigation, I have come to the conclusion that the various deposits are less frequent in the arteries most affected with aneurism, for example, the ascending aorta, and popliteal arteries (the most common seats of aneurism) are not oftener diseased than the descending thoracic, the lower part of the abdominal aorta, and the iliacs, where the disease is comparatively rare. I must therefore infer that these deposits are less concerned in the production of aneurism than is generally supposed, and that mechanical causes afford a more probable explanation." (pp. 114-15.)

Mr. Crisp finds that, including all the cases of aneurism reported in his table, the proportion of females is rather less than one eighth. This is no doubt owing to the less laborious nature of their occupations, and their being less exposed to mechanical injury. This proportion, however, is not maintained in particular aneurisms. Thus, for instance, in the 245 aneurisms of the aorta, pulmonary, and cerebral arteries, it amounts to about a fifth. Of the 25 carotid aneurisms, 13 occurred in males, 12 in females. In 308 cases of external aneurism, including the carotid, there were 276 males to 32 females, the latter forming barely a tenth; whereas in the 21 cases of dissecting aneurism there are 14 females to 7 males.

The period of life at which aneurism is most common, is between the ages of 30 and 40. Of the 551 cases reported in the table, 198 occurred between these ages: between 40 and 50 there were 129 cases. Whereas, before the age of 10, there was only one case, between 10 and 20, five cases, and after 70, eleven cases.

Mr. Crisp, in opposition to the opinions of many of the older writers on aneurism, and of some of the moderns, more particularly Mr. Porter, has not been able to satisfy himself that mercury exercises any influence in the production of this disease; he thinks, however, that syphilis, venereal excesses, the use of spirituous drinks, or any causes, mental or physical, producing violent and irregular action of the heart and arteries, must predispose to this affection. Of all the exciting causes of aneurism, whether external or internal, he believes violence and sudden muscular efforts to be the most frequent.

Mr. Crisp believes that aneurism is of more frequent occurrence in England than in any other country. This he attributes to the habits of life of the people, their great industry, the violent and continued exertions they are obliged to make, as well as their greater addiction to the use of ardent spirits. Sailors and soldiers are more liable to aneurism than any other class of the community, being often called upon to make sudden and very violent efforts. Amongst agricultural labourers, on the other hand, the disease is not frequent. Judging from the number of cases of aneurism reported in medical journals and publications, the only means that Mr. Crisp has been able to make use of in instituting a comparison between the frequency of the disease in this and other countries, he believes that next to England the disease is more prevalent in the United States of America, than in France, and, lastly, in Italy. It would appear from information that he has received from resident surgeons, that aneurism is of extremely rare occurrence both in the East and West Indies, and South America.

*Internal aneurism.* By internal aneurism, Mr. Crisp means those cases that are not within the reach of surgical operation. Of 243 cases of this description, 175 were in the thoracic aorta, 59 in the abdominal and its branches; 2 in the pulmonary artery, and 7 in the cerebral; 198 were males and 45 females. Three of these cases occurred before the age of 20; twenty-three between the ages of 20 and 30; eighty-two between 30 and 40; after this age the number of cases gradually diminished. In his account of the symptoms and diagnosis of this class of cases, Mr. Crisp adds nothing to what is already well known.

*Pathological effects of thoracic aneurism.* On this subject the analysis of the table of cases has furnished some very interesting and important results. Aneurism of the thoracic aorta may burst into the left pleura and posterior mediastinum; the former mode of termination is most generally considered of most frequent occurrence in these cases. From this opinion, however, Mr. Crisp dissents, and states that it is his belief that the most frequent mode of termination of aneurism of the descending aorta is by rupture into the pericardium. The tumour rarely bursts externally, although in some cases before death small discharges of blood take place; and death, from external hemorrhage, appears inevitable. Sometimes the internal tumour is considerably reduced in size before death, and in five instances it disappeared entirely. The following is the analyses of the mode of termination in all the cases of thoracic aneurism collected by Mr. Crisp.

"*Ascending aorta.* Of 98 cases of aneurism of the ascending aorta (76 males and 22 females) the terminations were as follow:—30 opened into the pericardium, 6 externally, 4 into the left pleura, 1 into the right, 3 into the pulmonary artery, 3 into the right lung, 3 into the superior cava, 2 into the œsophagus, 2 into the right auricle, 2 into the right ventricle, 1 into the left, 1 into the trachea; 7 died from pressure on the trachea and bronchi, 6 from serous effusion into the pericardium and pleura, 2 from phthisis, 2 from syncope and exhaustion, 2 from coma, 1 from each of the following—regurgitation into the ventricle, compression of vena cava, of pulmonary artery, cerebral congestion, apoplexy of lung, distension of stomach, pericarditis, bronchitis, and hemoptysis; 4 died suddenly, without rupture of the sac, and in 8 the cause of death was doubtful.

"*Aortic arch.* In 48 cases of aneurism of the arch of the aorta (35 males and 13 females), 4 opened into the trachea, 2 into the pericardium, 2 into the œsophagus, 2 into posterior mediastinum, 2 into right pleura, 2 into the left, 2 into the bronchus, 1 into the pulmonary artery, 1 into the superior cava, 1 into the lung, 1 externally; 12 died from supposed pressure on the trachea and bronchi, 3 from suffocation, 2 from hydrothorax, and 1 from each of the following—pressure on the recurrent laryngeal nerve, pressure on the cava, hæmoptysis, dysentery, serous apoplexy, rupture of the descending aorta, and external violence; and in 6 cases the cause of death is not stated.

*Descending thoracic.* Of the 21 cases of aneurism of the descending aorta (all occurring in males), 4 opened into the right pleura, 4 into the left, 5 into the œsophagus, 2 into the left bronchus, and 1 into the left lung; 2 died from pressure on the trachea and left bronchus, 1 from exhaustion and suffocation, 1 from apoplexy; 8 cases are described as aneurism of the thoracic aorta, and their termination not given.

*Treatment.* In the treatment of internal aneurism little can be done towards effecting a cure, but in many cases much may be accomplished towards alleviating the sufferings and prolonging the life of the patient. Our author lays some stress on the importance of small and repeated bleedings in these cases, with the view of diminishing the impulse of the left ventricle, and consequently the force with which the aneurismal sac will be distended. Six or eight ounces of blood taken at regular intervals he has found of much service, both as regards the diminution of pain and the prolongation of life. When much pain is present the preparations of opium will afford most relief, and in some cases large doses of this medicine will be required. Sprinkling a blistered surface with the muriate of morphia has also been productive of some benefit. The diet is a matter of much importance in the treatment of aneurism; it should be as simple as possible, and all nourishment of a stimulating character should be avoided, on the principle that anything that produces increased action of the heart is injurious.

*Aneurism of the abdominal aorta.* Of fifty-nine cases of aneurism of the abdominal aorta, recorded in the table, fifty-one were males and eight females. Eleven were between the ages of 20 and 30; twenty-two between 30 and 40; fourteen between 40 and 50; and three between 50 and 60. The ages of nine are not given. As the abdominal viscera are not so essential to life as the thoracic, these aneurisms may attain an immense size before proving fatal. In Mr. Langstaff's museum was one, the coagulum in which weighed five pounds and a half. In the Chatham museum is one, the coagulum of which weighed ten pounds; and in the museum of the London Hospital is an abdominal aneurism, the sac of which held just one pailful of water.

Tumours of various kinds in the abdomen, whether solid or fluid, may be mistaken for aneurism of the abdominal aorta; but what is called nervous pulsation of the aorta, is by far the most frequent source of error, and it often requires much nicety to distinguish between the two diseases. The following are the diagnostic signs given by our author.

"In abdominal aneurism, pain of an aching, tearing, or darting character is often felt. In aortic pulsation the patient seldom complains of pain in the immediate neighbourhood of the aorta. The sound, when heard in abdominal aneurism, is generally a single rough bruit. In aortic pulsation abnormal sound is seldom present. The best diagnostic marks are the following: in abdominal aneurism the impulse is mostly heaving and confined; in aortic pulsation free and bounding. The dissection of the sac in abdominal aneurism is generally lateral or posterior, and the tumour is seldom distinctly felt, when seated near the cæliac axis. The state of the abdominal viscera, the sex, age, habits, and constitutional peculiarities of the patient, are circumstances which must not escape the notice of the medical attendant.

"Abdominal aneurism more frequently commences near the cæliac axis, where (as I have before observed) the arterial tunics are probably much weakened by the origin of the collateral branches; the diameter of the vessel is likewise very perceptibly diminished below this part. In several instances recorded in the Table of Aneurisms, rupture of the sac took place some time before death, the blood finding its way behind the peritoneum. At the time of rupture the pain is very acute from the detachment of this membrane from its cellular connexions; there is also great depression of the vital powers. After a few hours or days the peritoneum gives way and the blood escapes into its cavity." (p. 163.)

The mode in which the fifty-nine cases of aneurism of the abdominal aorta and its branches collected by Mr. Crisp, terminated, is as follows:—

Eleven opened behind the peritoneum, ten into the peritoneum, four into the left pleura, three into the vena cava inferior, one into the lung, one into the colon, one into the pelvis of the kidney, one into the posterior mediastinum, one into the chest, two died suddenly without rupture of the sac, two from jaundice, and one from each of the following causes: —heart disease, scarlatina, phthisis, diarrhœa, exhaustion, hydrothorax, anasarca and ascites, senile gangrene, and rupture of sac by vomiting. The terminations of twelve cases are not given.

*Cerebral aneurism.* The cerebral arteries are, according to Mr. Crisp, more frequently the subject of aneurismal dilatation than others of a similar size. This he attributes to their unsupported state from the absence of a cellular sheath, the frequency of concussions of the skull, and the irregularity of the circulation in the brain from mental or bodily excitement. The eight cases of cerebral aneurism that are reported in the table all occurred in males. Five of the aneurisms were seated in the basilar, three in the cerebral arteries. They varied in size from a pea to a common apple. Three of the patients were under twenty years of age, and the remaining five between thirty-five and sixty. In the three that were seated in the cerebral arteries, death took place by rupture of the sac and extravasation of blood; in the remaining five, those seated in the basilar artery, sanguineous apoplexy, cerebral congestion, encephalitis, hemiplegia, and pressure on the medulla oblongata were the causes of death.

*Dissecting aneurism.* The following is the condensed account that our author gives of his views on dissecting aneurism.

"This variety of aneurism is much more frequent in women than in men, and most of the patients are advanced in years. It is not difficult, I think, to understand why this form of the disease should occur more frequently in the aged, when we consider the state of the arterial tunics at this period of life, their connecting media, from increased dryness, being more readily destroyed. I have performed several experiments on the coats of arteries of persons of different ages, which have fully confirmed this opinion. Thus, if a small syringe, Anel's, is filled with water, and its nozzle inserted into the layers of the fibrous coat, or between it and the cellular coat of an artery taken from an old subject, the fluid will be rejected a considerable distance between the tunics. If, however, the experiment be made on a young subject, considerable difficulty will be experienced in separating the laminæ, and the water will only divide the coats to a small extent. I am, however, at a loss to account for the greater frequency of this disease in females, and I will not at present attempt an explanation. The lower part of the ascending aorta is the most common seat of these ruptures, and the blood is generally discharged into the pericardium. Thus, 15 cases terminated in this manner, and 4 died of the following—senile gangrene, tuberculated lungs, dropsy, and orthopnœa; in 2 the cause of death is not given. This lesion is generally attended with acute pain, great collapse, sense of suffocation, and constriction of the chest; there are no positive signs, however, which can indicate its existence. In some cases death takes place at the time of the rupture; in others the cellular coat of the aorta and its pericardial covering form a barrier for a few days to the passage of the blood into the pericardium; whilst in a third variety a new channel is formed by the splitting of the coats; and in one instance this channel appeared to be lined with false membrane, the patient living for a considerable time after the rupture. Seven of the patients died suddenly, seven lived from one to twelve days after the accident; another survived a month; another lived several months; and in a third the period is not named. The coats may be separated on one side of the artery only, or the splitting may extend to the whole circumference of the vessel. In some instances, the blood, after dissecting the coats, again found its way into the artery; in two cases only did the rupture occur in the abdominal aorta, and in three others the blood passed along the coats of the thoracic to the abdominal aorta, being discharged above into the pericardium. From the experiments I have made I am disposed to think that an artery, containing a large quantity of bony and atheromatous matter, is not so likely to be affected with this form of aneurism, as one which is nearly free from deposit, the adventitious substance tending rather to prevent the splitting of the coats." (p. 168.)

*External aneurism.* We shall now give an analysis of the statistical data that Mr. Crisp has collected on the subject of external aneurism, in reference to the comparative frequency of this disease in different arteries and at different ages, and in the results of this ligature.

The observations made by our author on the general treatment of external aneurisms, on their spontaneous cure, and on the employment of pressure in those cases to which it is applicable need not detain us, as he gives nothing with which the profession is not already familiar. Indeed his observations on the employment of pressure in the cure of aneurisms, a subject of the greatest interest, and one to which the attention of surgeons is peculiarly directed at the present time, are very incomplete, evincing less acquaintance with the principles on which the modern treatment by pressure is conducted than we could have expected. Mr. Crisp's account contrasts very unfavorably with the very excellent and practical Essay lately published on this subject by Dr. Bellingham of Dublin, to which we would refer our readers as containing a most masterly account of the treatment of aneurism by compression. Mr. Crisp's observations on another mode of treatment that is also beginning to attract some attention, both in this country and abroad—we allude to the galvano-puncture—are also far too cursory and superficial. Short as they are, however, they are not free from error, the introduction of this plan of treatment being attributed to M. Petrequin of Lyons, instead of to a countryman of our own, Mr. Benjamin Phillips, who was the first to direct the attention of the profession to this subject, in an essay published as far back as the year 1832.

*Secondary hemorrhage.* The statements of different observers vary considerably in respect to the frequency of this accident after ligatures. Lisfranc states as the result of his inquiries into the details of 180 operations for aneurism performed according to the Hunterian method, that secondary hemorrhage occurred in 32; being in the ratio of 1 in 6. Porta in his recent work on the Ligature of Arteries, has collected 600 cases of the ligature of the principal arteries, in 75 of these secondary bleeding occurred; of these 31 were cured, 30 died from loss of blood, and 14 from other accidents. The treatment adopted in 31 cases was as follows: the plug was used in 13, torsion in 4, a second or third ligature in 9, bandaging or cold applications in 3, and amputation of the limb in 2. It will be seen by this, that according to Porta, secondary hemorrhage only occurs in about one twentieth of the cases, instead of in one sixth, as stated by Lisfranc. This discrepancy may very probably be reconciled by the fact that Lisfranc has only included in his estimate cases in which the vessel has been ligatured for aneurism, whereas Porta speaks of the results of the ligature for wounds as well as for disease. Mr. Crisp finds that of 256

cases of ligature of the larger arteries for aneurism, 21 patients died of hemorrhage, and in 8 cases the termination was of a mixed character, but bleeding to a certain extent had occurred, and therefore had some influence in producing the fatal result. The deaths lately from hemorrhage amounted to rather more than one twelfth, including the doubtful cases to barely a ninth; whilst the general mortality is 22 per cent., the general mortality of Porta's cases being 27 and a fraction per cent. All these operations were for the cure of spontaneous aneurism, and cases of the distal operation, of ligature of the aorta, the innominata, and of the subclavian, on the anterior side of the scalenus are included. If these cases were omitted, the rate of mortality would be considerably diminished.

The following is the time of separation of the ligatures in 150 cases of operation for the cure of aneurism, nearly all of them spontaneous. In two cases of ligature of the common iliac, the ligatures separated on the 18th and 35th days; of the internal iliac, on the 21st and 42d days. In 33 cases of ligature of the external iliac, on an average on the 22d day. In 54 cases in which the femoral was tied, the ligature remained on an average 18 days. In 30 cases of ligature of the subclavian, 17 days; in 21 cases of ligature of the carotid, 21 days; and in 8 cases of the brachial, 14 days.

*Aneurism of the innominata.* Of 20 cases of aneurism of the innominata, 15 of the subjects were males, and 5 females. Aneurism of this vessel seldom burst internally,—not one of the 20 cases terminating in this manner;—1 burst externally, 3 proved fatal by pressing on the trachea, 1 on the right bronchus. Serous effusion into the pleura, pericarditis, and exhaustion carried off 3 others; 6 were subjected to surgical operations, and in 5 the cause of death is uncertain. The application of the ligature to this artery has, in every case, been unsuccessful, and few surgeons would, we think, at the present day be found who would venture under any circumstances to have recourse to this operation. There are only three instances of cure of aneurism of the innominata on hand. This occurred to Mr. Porter, who attempted to tie the artery, but on cutting down upon it, he did not think it safe to apply the ligature; strange to say, the patient recovered, pulsation ceasing entirely in the sac. Another case was treated successfully by Mr. Luke, by small bleedings frequently repeated, and digitalis; and the third was a case in which the right carotid was tied by Mr. Evans; the patient was well nine years after the operation.

*Aneurism of the subclavian.* Twenty-six cases of aneurism of this artery are on record; of these 25 were males, and only one a female. 15 occurred in the right, and 8 in the left subclavian; in 3 the situation is not mentioned. The artery was ligatured in 23 of these 26 cases; of these operations 11 were successful, and 12 terminated fatally. In 4 of these unsuccessful cases, however, the artery was tied internal to the scalenus; in 2 the innominata was ligatured, and in 2 the sac was punctured, so that if these eight cases be included, the mortality from ligature of this artery when the operation is performed at an early period of the disease, and in the third stage of the vessel, is not very great. We may mention that all the cases in which the vessel has been tied internal to the scalenus have terminated fatally.

The results of the ligature of the subclavian artery for axillary aneurism form a striking contrast with the preceding statement. Of 14 cases in which the ligature was applied to the subclavian for this disease, 11 were successful.

*Carotid aneurisms.* Of twenty-five cases of this disease, thirteen were males and twelve females. The frequency of the disease amongst females is a remarkable fact which we have not seen noticed by any other writer, and which Mr. Crisp attributes to the exposed state of the neck of females, and its consequent liability to injuries. Of these 25 cases, 21 were operated upon. Ten of these operations were successful, and eleven had an unfavorable issue. Five of the operations were performed on the distal side of the sac; of these three were successful, and in two the life of the patient was prolonged. The principal sources of danger after the ligature of this vessel are, as Dr. Miller and Dr. Norman Chevers have shown, referable to engorgement of the lungs or to cerebral disturbance, and though Mr. Crisp doubts the accuracy of Dr. Chevers's conclusions with regard to the frequency of the cerebral disturbance after ligature of the common carotids, there remains in our minds no doubt of the fact, after perusing the evidence that he has adduced in support of it.

*Iliac arteries.* Only two cases of aneurism of the common iliac are mentioned in the table; both occurred in males: one was a varicose aneurism, in the other the aorta was tied.

Aneurism of the external iliac occurred in nine patients, all males. In two cases the common iliac was tied: in the first, death occurred on the eighth day, from hemorrhage; in the last, the operation was successful; and in four it was ligatured successfully.

With the exception of the popliteal, the upper third of the femoral artery is the most frequent seat of external aneurism. Mr. Crisp has collected 66 cases of aneurism in this situation, 61 of which occurred in males and but 5 in females. A remarkable fact connected with this species of aneurism is its frequency in sailors: of 39 patients whose occupations are given, no less than 12 being sailors. In these 66 cases the external iliac was ligatured 43 times, in two instances the femoral was tied also; 36 of these cases terminated favorably; of the 10 unsuccessful, 3 died from hemorrhage; 2 from sloughing of the sac; 2 from pressure; and 1 from gangrene; another from tetanus, and a third without apparent cause. The femoral artery alone was tied in 12 cases: of these 9 were successful, and in 5 cases amputation was practised with success.

*Popliteal aneurism.* The table contains 137 cases of aneurism of the popliteal artery. Of these, 133 were males and 4 females; in 44 cases the left side was affected; in 40 the right, in 42 the situation is not named; and in 11 cases the aneurism was double. Of these 137 cases, 119 were cured and 18 died. In 110 cases the Hunterian operation was performed in 91 with complete success, and 7 others terminated favorably after amputation, the ligature having been previously used. The 11 patients affected with double popliteal aneurisms were all cured, the ligature being used in 10 and pressure in one.

*Circoid aneurism, or aneurism by anastomosis.* Forty-five cases of this disease are arranged in a tabulated form: 24 of these were males, 16 females, and the sex is not stated in 5 cases. In 38 cases the disease was seated in the head, neck, and upper extremities. The lower part of the rectum, perineum, nates, scrotum, leg, knee, and foot, were affected in the remaining seven; 22 were cured, 6 benefited, 11 died after the operation, and 4 were not operated on. In 16 cases of this disease, seated about the head and face, the carotid was ligatured; and in 3 patients both carotids were tied. The ligature alone was successful in 5 cases; in 7, benefit was derived; and the disease was afterwards removed by pressure, caustic, or incision. In the 3 fatal cases, the patients did not appear to die from the immediate effects of the operation.

Our author next devotes a page or two to some cursory remarks on the important and interesting subjects of aneurism of bone and fungus hæmatodes, and gives two or three short chapters on ruptured, torn, and wounded arteries, which, as they are not the result of personal observation, need not detain us; these are followed by a mere sketch of the diseases of the veins, with which the work concludes.

On the whole, we regard Mr. Crisp's book as a valuable addition to our surgical literature. Our remarks on various parts of it show that we think it might have been made better than it is; but, with all its imperfections, we consider it as highly creditable to its author. It can have no pretensions to be considered a *complete* treatise on the diseases of the blood-vessels, many of the most important of these affections being treated only in a superficial and incomplete manner. Its principal value consists in the Table of Aneurisms which the author has, with great industry and care, constructed from various sources; and by the analysis of which new light is thrown on several points connected with aneurism, and the result of the ligature of arteries. The value of this collection would, however, have been much increased had the cases been arranged in a systematic manner, and not put down merely in the order in which the author met with them, without any attempt at classification or arrangement. If called on for a new edition, Mr. Crisp will add greatly to the value of his work by enlarging his statistics still further, so as to include the records of foreign as well as of British pathology and practice; and by paying more attention to some of the points on which we have taken the liberty to remark.

---

